# Balloon-assisted enteroscopy-ERCP with percutaneous transhepatic rendezvous technique for placement of a self-expanding metal stent

**DOI:** 10.1055/a-2119-0875

**Published:** 2023-07-27

**Authors:** Alvaro Martínez-Alcalá García, Frank Aedtner, Klaus Mönkemüller

**Affiliations:** 1 Centro de Inovaciones Digestivas CIDMA, Sevilla, Spain; 2 Division of Endoscopy, Ameos Teaching University Hospital, Halberstadt, Germany; 3 “Prof. Carolina Olano” Division of Gastroenterology, Universidad de La República, Montevideo, Uruguay; 4 Department of Gastroenterology, Virginia Tech Carilion School of Medicine, Virginia, USA

Endoscopic retrograde cholangiopancreatography (ERCP) in patients with surgically altered upper gastrointestinal anatomy is challenging. For these patients, balloon-assisted enteroscopy (BAE)-ERCP has been shown to be feasible, safe, and effective 1 . However, only plastic stents can be placed through the working channels of enteroscopes 2 . Here we present the concept of double-balloon endoscopy (DBE)-assisted-ERCP rendezvous technique with combined endoscopic–percutaneous placement of a biliary self-expanding metal stent (SEMS).

A 45-year-old man with a history of autoimmune hepatitis who had undergone liver transplantation with Roux-en-Y hepaticojejunostomy presented with cholangitis and a hepaticojejunostomy stricture. A percutaneous transhepatic cholangiodrain (PTCD) had been placed to relieve the bile duct stricture but there were also bile duct stones ( Fig. 1 ). Thus, BAE-ERCP was performed to attempt removal of the bile duct stones. The double-balloon endoscope was advanced to the afferent limb where the PTCD was seen exiting the hepaticojejunostomy ( Video 1 ). Due to massive looping of the endoscope it was impossible to advance any balloons, baskets, or stents through the scope ( Fig. 1 ). Therefore, it was decided to first dilate the hepaticojejunostomy and then place the endoscopic stent from outside, i. e. percutaneously, under endoscopic view. A biliary wire was advanced percutaneously into the jejunum across the hepaticojejunostomy. The PTCD was then removed. The dilating balloon was advanced from outside and dilation was performed under both direct endoscopic and fluoroscopic visualization ( Fig. 1 , Video 1 ). All the stones and sludge were removed. Then one 8- ×  60-mm fully covered SEMS was inserted over the wire and successfully released ( Video 1 ). The patient had an uneventful recovery and no more pain at the ex-PTCD site. The SEMS was removed 6 months later with complete resolution of the stenosis of the hepaticojejunostomy.

**Fig. 1:**
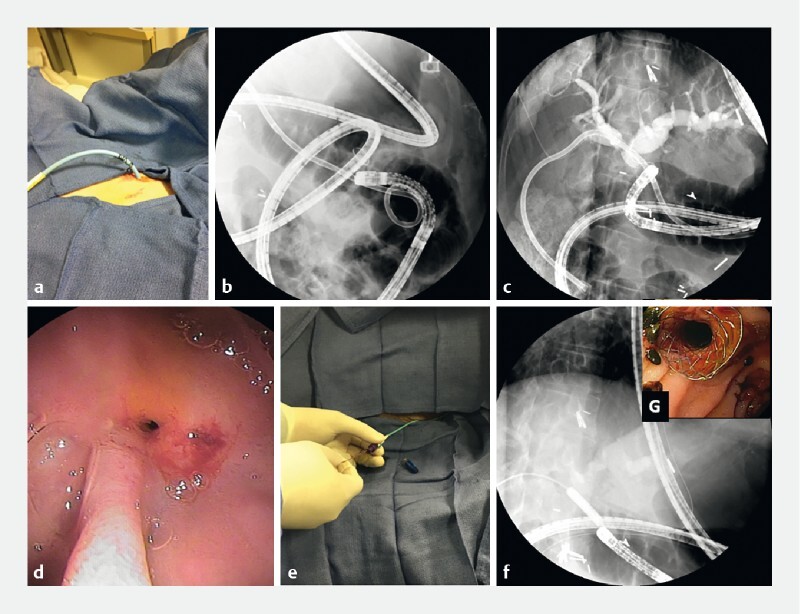
Double-balloon enteroscopy-assisted endoscopic retrograde cholangiopancreatography rendezvous technique with combined endoscopic–percutaneous placement of a self-expanding biliary stent. **a** Percutaneous transhepatic cholangiodrain (PTCD) in place. **b** Massive looping of the endoscope made it impossible to advance any balloons, baskets, or stents through the scope. **c** Cholangiogram shows dilated bile ducts and hepaticojejunostomy stricture. **d** The hepaticojejunostomy was very stenotic. **e** Biliary guidewire being placed through the PTCD. **f** The stricture was dilated with a CRE balloon (Boston Scientific). **g** Endoscopic view of fully covered self-expanding metal stent inserted into the bile duct across the hepaticojejunostomy.

**Video 1** Double-balloon enteroscopy-assisted endoscopic retrograde cholangiopancreatography rendezvous technique with combined endoscopic–percutaneous placement of a self-expanding biliary stent.


Our case shows the steps for solving a complex postoperative situation. If the patient has a percutaneous stent, then the BAE-ERCP rendezvous technique presented herein can be attempted, resulting in successful placement of larger-diameter plastic stents and/or SEMS which cannot be advanced through any enteroscope, diagnostic or therapeutic, even when the endoscope is not torqued.

Endoscopy_UCTN_Code_CCL_1AZ_2AK

## Notes

Endoscopy E-Videos

https://eref.thieme.de/e-videos
*E-Videos* is an open access online section of the journal *Endoscopy* , reporting on interesting cases and new techniques in gastroenterological endoscopy. All papers include a high-quality video and are published with a Creative Commons CC-BY license. Endoscopy E-Videos qualify for HINARI discounts and waivers and eligibility is automatically checked during the submission process. We grant 100% waivers to articles whose corresponding authors are based in Group A countries and 50% waivers to those who are based in Group B countries as classified by Research4Life (see: https://www.research4life.org/access/eligibility/ ). This section has its own submission website at https://mc.manuscriptcentral.com/e-videos
